# Supplementary material for: Integrating single-cell RNA sequencing and prognostic model revealed the carcinogenicity and clinical significance of FAM83D in ovarian cancer
Source: Front Oncol. 2022 Dec 8;12:1055648. doi: 10.3389/fonc.2022.1055648 (PMC9773999; doi:10.3389/fonc.2022.1055648)
Supplement: Supplementary file 1 [file DataSheet_1.docx]

Supplementary Material

**Supplementary Tables and Figures**

- 1. **Supplementary Tables**

**Supplementary Table 1. Gene primer sequence**

| Gene symbol | Primer sequence (5'--3') |
| --- | --- |
| FAM83D-F | ATGACAGTTCGGACTATCACAGG |
| FAM83D-R | ACTTTTCGTGAACCTTCCCAAT |
| E-cadherin-F | CGAGAGCTACACGTTCACGG |
| E-cadherin -R | GGGTGTCGAGGGAAAAATAGG |
| N-cadherin -F | AGCCAACCTTAACTGAGGAGT |
| N-cadherin -R | GGCAAGTTGATTGGAGGGATG |
| Vimentin-F | GACGCCATCAACACCGAGTT |
| Vimentin -R | CTTTGTCGTTGGTTAGCTGGT |
| GAPDH-F | GGAGCGAGATCCCTCCAAAAT |
| GAPDH-R | GGCTGTTGTCATACTTCTCATGG |

**Supplementary Table 2.** **All the datasets used in the manuscript.**

1. Clinical information of two single-cell sequencing samples

| pathologic diagnoses | | Age(y/o) | | | Race | Stage | Tumor Marker |
| --- | --- | --- | --- | --- | --- | --- | --- |
| High-grade serous carcinoma of ovary with omental metastasis, lymph nodes metastasis, appendiceal and gastric surface metastases | **57** | | | ethnic Han | | IIIC | CK7^+^  WT1^+^  PAX8^+^  PR^+^ |
| normal ovary control | | | **26** | | ethnic Han | **-** | **-** |

1. The Gene Expression Omnibus (GEO) OC datasets

| GSE ID | Samples | Platform | Download |
| --- | --- | --- | --- |
| GSE18520 | 10 normal samples and 53 OC samples | Affymetrix HG-U133 Plus 2.0 (GPL570) | GEO database (https://www.ncbi.nlm.nih.gov/) |
| GSE9891 | 285 ovarian samples | Affymetrix HG-U133 Plus 2.0 (GPL570) | GEO database (https://www.ncbi.nlm.nih.gov/) |
| GSE45553 | 4 cisplatin-resistant and 4  cisplatin-sensitive samples | Affymetrix Human Gene 1.0 ST Array (GPL6244) | GEO database (https://www.ncbi.nlm.nih.gov/) |

|  |  |  |  |
| --- | --- | --- | --- |
|  |  |  |  |

1. The Cancer Genome Atlas (TCGA) OC datasets

| Data | Samples | Download |
| --- | --- | --- |
| RNA-seq | 308 | UCSC Xena (https://xenabrowser.net/datapages/) |
|  |  |  |

1. Kaplan-Meier plotter OC datasets

| Samples | Survival | Download |
| --- | --- | --- |
| 1656 | OS | http://www.kmplot.com |

1. Oncomine datasets

| Data | Samples | | Download |
| --- | --- | --- | --- |
| Lu Ovarian cancer | | 5 normal samples and 45 tumor samples | http://www.oncomine.org |
| Yoshihara Ovarian cancer | | 9 normal samples and 43 tumor samples | http://www.oncomine.org |
|  |  | |  |

**1.2 Supplementary Figures**


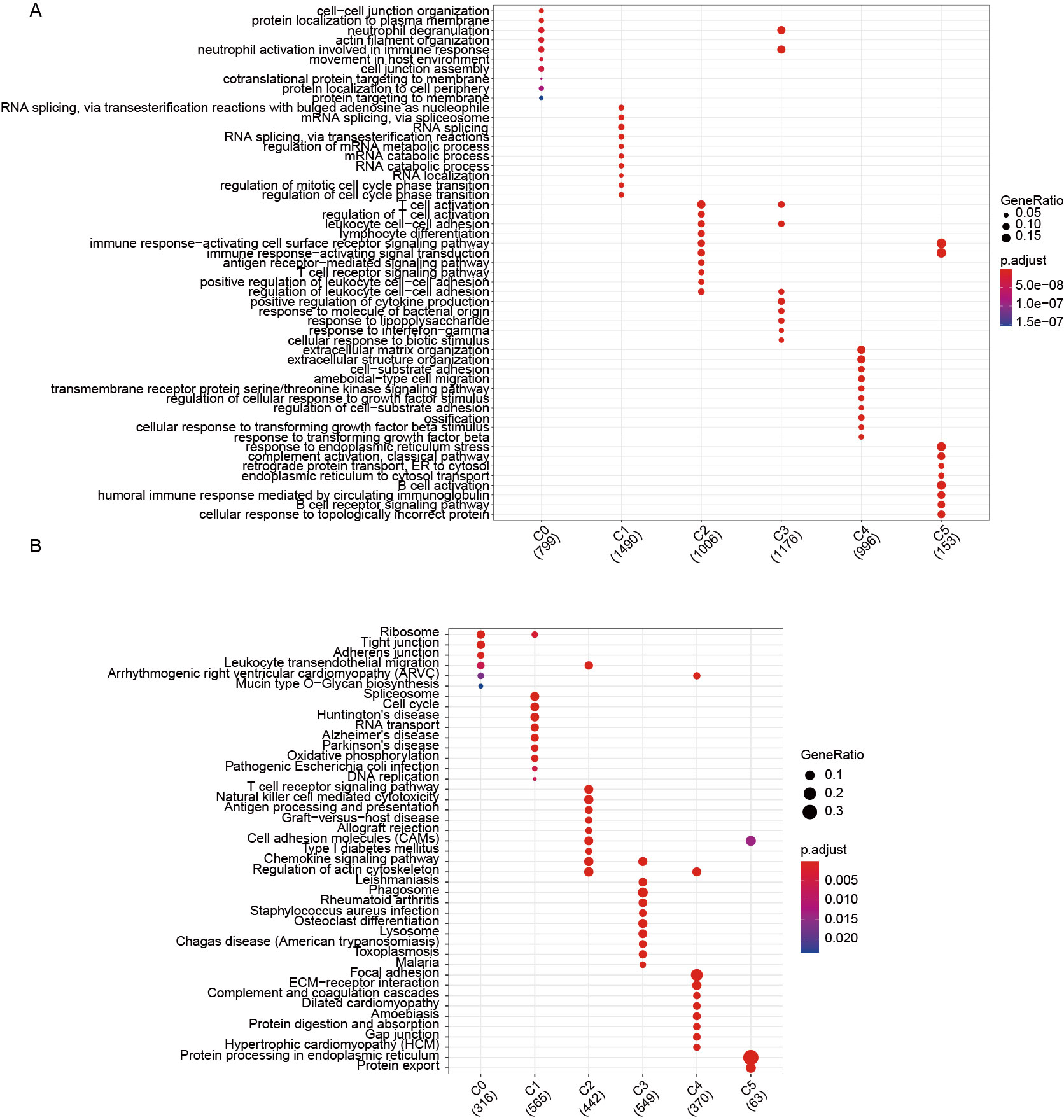


**Supplementary Figure 1. Gene Ontology (GO) terms and KEGG pathway analysis of diverse cell types.**

1. Gene Ontology (GO) terms analysis of C0-C5.
2. KEGG pathway analysis of C0-C5.


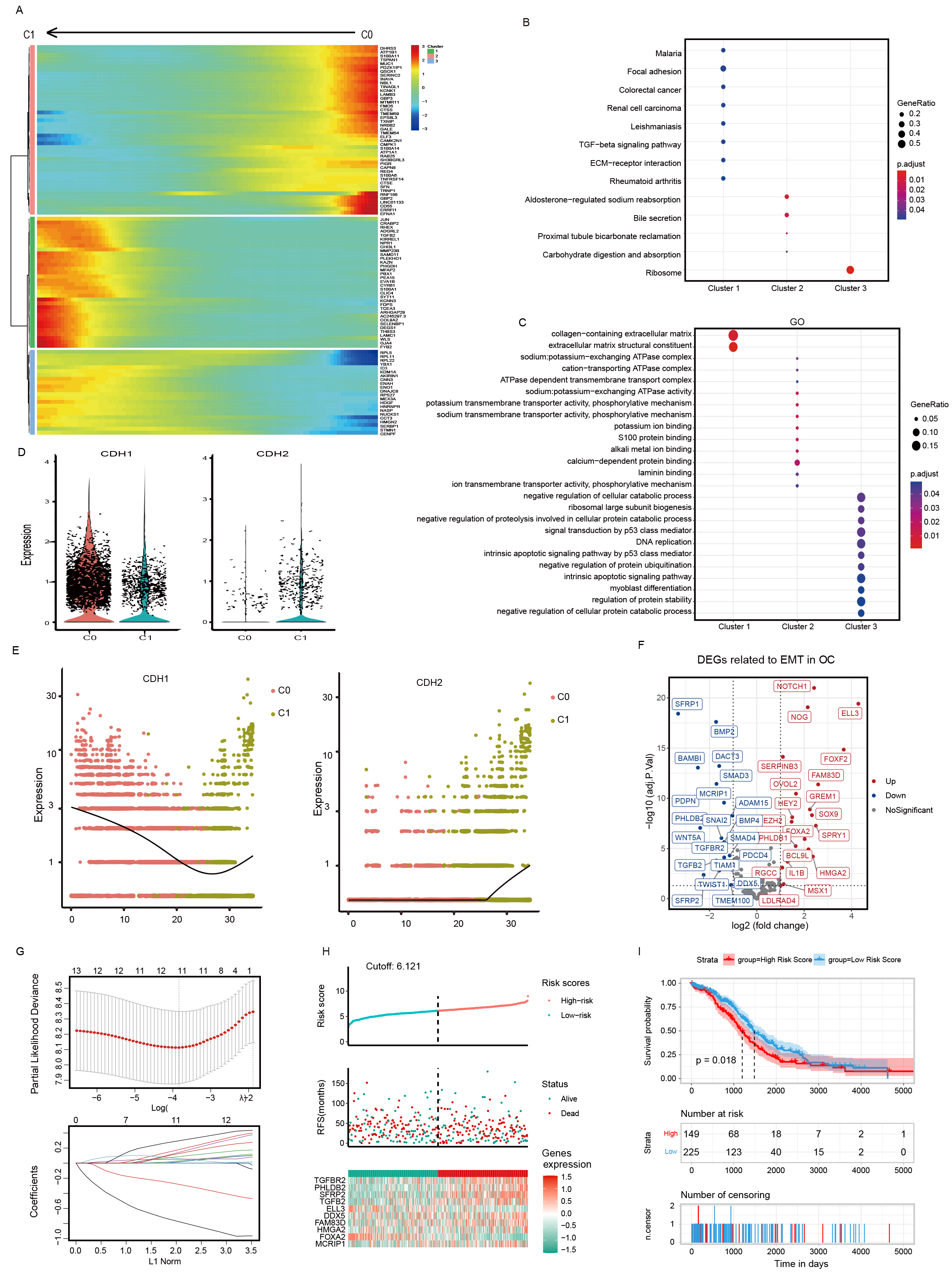


**Supplementary Figure 2. C1 has excessive EMT characteristics.**

1. The heatmap displayed the SDE genes during progression from C0 to C1.

(B and C) The KEGG and GO analysis revealed enriched functions of the SDE genes.

(D and E) Pseudo- time analysis and expression of EMT key genes in C0 and C1.

(F) DEGs related to EMT in OC.

(G) The lasso coefficient section. It shows that the tuning parameters were selected in the lasso model through 10x cross validation according to the minimum standard of the operating system(λ); Draw a vertical dashed line at the best value using the minimum value and the 1-se standard.

(H) Risk score distribution, survival status and gene expression profile of patients in high-risk score group and low-risk score group in test cohort.

(I) Kaplan Meier curve of OS in high-risk group and low-risk group of test cohort.


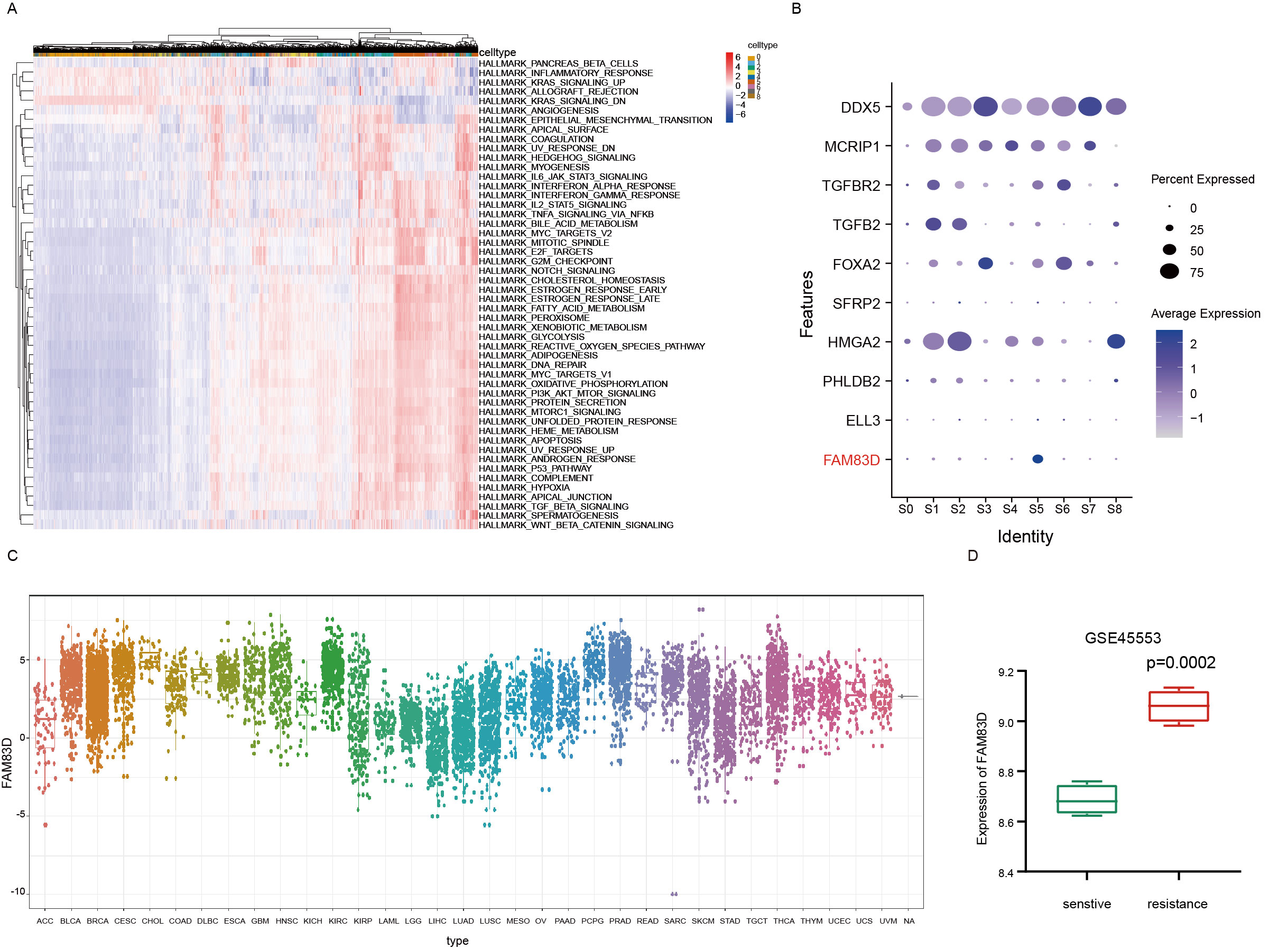


**Supplementary Figure 3. S5 was associated with multiple carcinogenic pathways, and the specific marker FAM83D was highly expressed in a variety of tumors.**

1. The Heatmap revealed the different enrichment of HALLMARK pathway in S0-S8.
2. The dot plot of 10 genes in malignant epithelial cell subgroups.

(C) FAM83D was highly expressed in ovarian cancer and other cancers in TCGA database.

(D) Box diagram showing higher FAM83D in cisplatin resistant group of GSE45553.


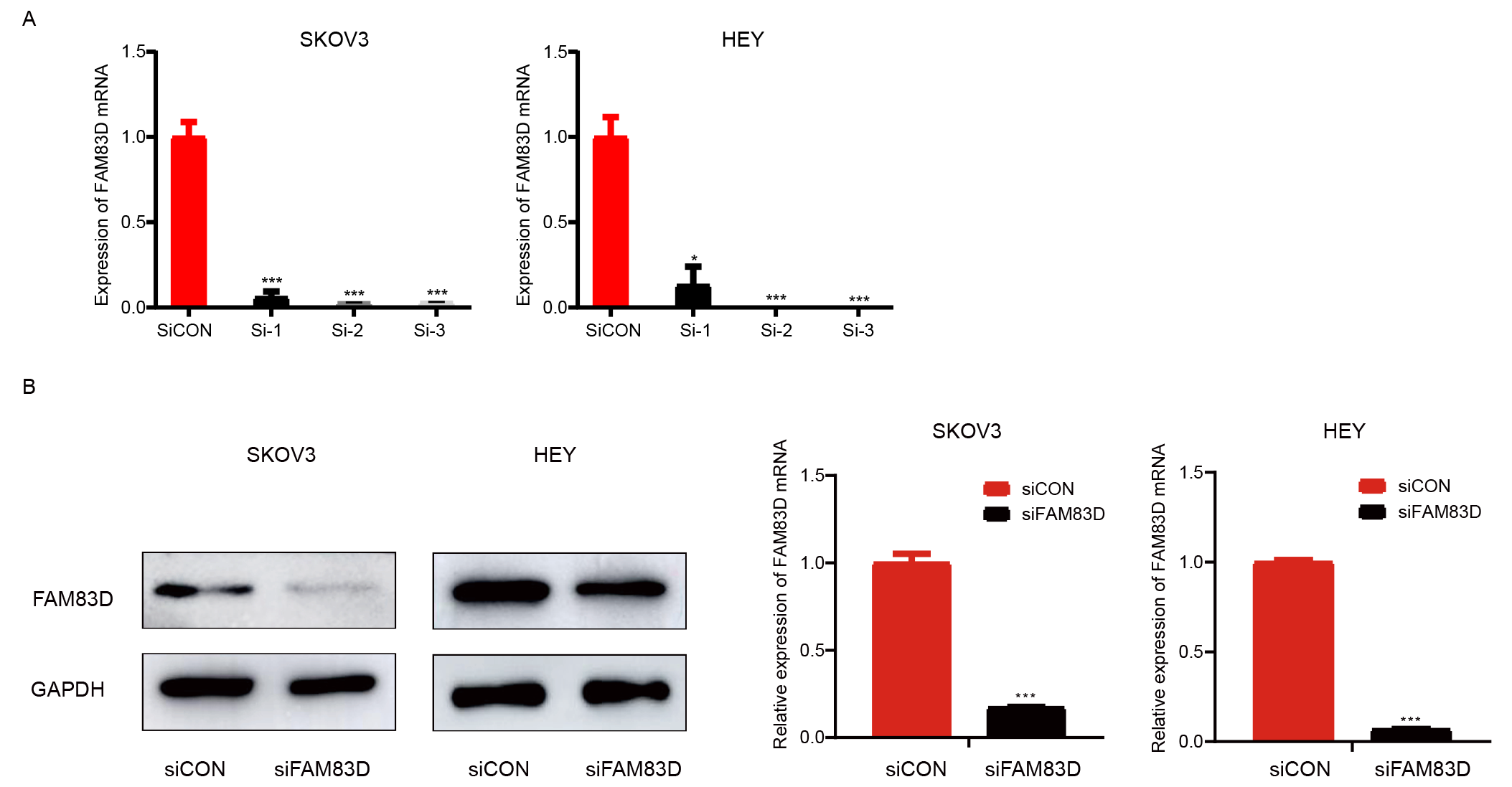


**Supplementary Figure 4. qRT-PCR and WB validated efficiency by using distinct siRNA to knockdown FAM83D.**

1. qRT-PCR analysis of FAM83D expression in OC cells transfected with siRNA. Cells were transfected with FAM83D siRNA2 and siRNA3 or control siRNA (JTS scientific) for subsequent experiments.
2. Western blot and qRT-PCR analysis of FAM83D expression in siCON and siFAM83D. ***p < 0.001 vs. cells transfected with siCON.


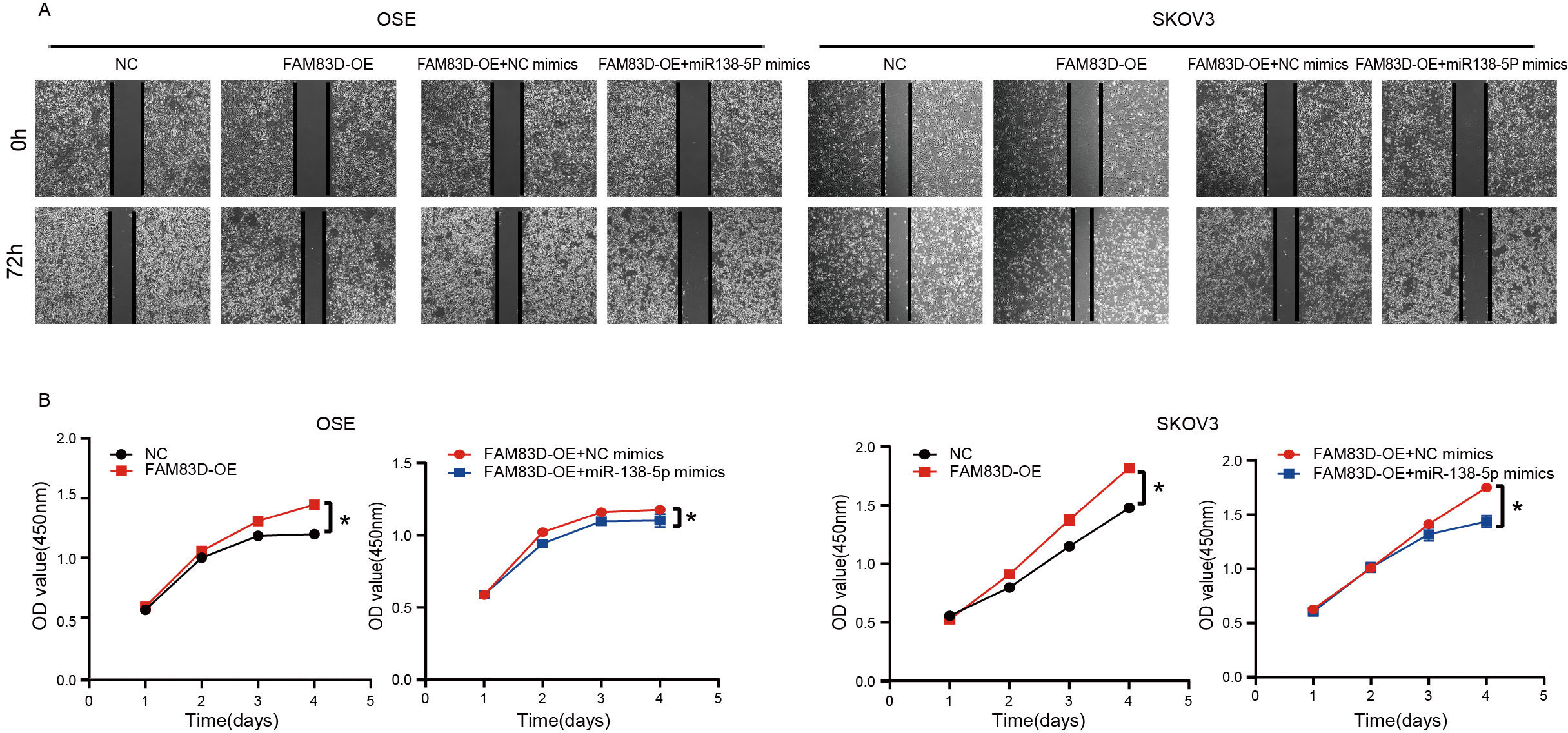


**Supplementary Figure 5. MiR138-5p alleviated the ability of FAM83D to promote ovarian cancer cell migration and proliferation**

1. Scratch wound healing showed the cell migration in OSE_FAM83D-OE_ and SKOV3_FAM83D-OE_ cells transfected with miR138-5p mimics. Representative images are shown.
2. CCK8 assay indicated the cell proliferation in OSE_FAM83D-OE_ and SKOV3_FAM83D-OE_ cells transfected with miR138-5p mimics.

*p<0.05 and **p<0.01 compared with NC.
